# An update on toxicology of aluminum phosphide

**DOI:** 10.1186/2008-2231-20-25

**Published:** 2012-09-03

**Authors:** Ali Akbar Moghadamnia

**Affiliations:** 1 Department of Pharmacology, Babol University of Medical Sciences, Babol, Iran

**Keywords:** Aluminum phosphide, Phosphine, Management, Poisoning, Suicide

## Abstract

Aluminum phosphide (AlP) is a cheap solid fumigant and a highly toxic pesticide which is commonly used for grain preservation. In Iran it is known as the “rice tablet”. AlP has currently aroused interest with increasing number of cases in the past four decades due to increased use in agricultural and non-agricultural purposesand also its easy availability in the markets has increased its misuse to commit suicide. Upon contact with moisture in the environment, AlP undergoes a chemical reaction yielding phosphine gas, which is the active pesticidal component. Phosphine inhibits cellular oxygen utilization and can induce lipid peroxidation. It was reported that AlP has a mortality rate more than 50% of intoxication cases. Poisoning with AlP has usually occurred in attempts to suicide. It is a more common case in adults rather than teen agers. In some eastern countries it is a very common agent with rapid action for suicide. Up to date, there is no effective antidote or treatment for its intoxication. Also, some experimental results suggest that magnesium sulfate, N-acetyl cysteine (NAC), glutathione, vitamin C and E, beta-carotenes, coconut oil and melatonin may play an important role in reducing the oxidative outcomes of phosphine. This article reviews the experimental and clinical features of AlP intoxication and tries to suggest a way to encounter its poisoning.

## Introduction

### Background

Aluminum phosphide (AlP) is a solid fumigant which has been extensively used since the 1940s. It is easily available and is purchased in some countries such as India under trade names e.g. Celphos, Quickphos, Synfume and Phosfume [1]. It has been registered in the USA and many other countries for the indoor fumigation of agricultural compounds, animal feeds, processed foods and also structural as well as outdoor pest control. In Iran it is known as the rice tablet [2] that can be purchased in local shops.

AlP is a solid pesticide that rapidly became one of the most commonly used grain fumigants because of its properties which are considered to be near ideal; it is toxic to all stages of insects, highly potent, does not affect seed viability, is free from toxic residues and leaves little residue on food grains [3]. This highly toxic chemical is cheap and usually formulated in tablets or pellets, granules and as a dust. Upon contact with moisture in the environment, it undergoes a chemical reaction yielding phosphine gas, which is the active pesticidal component [4]. It emerges as a poison of suicidal deaths as this pesticide has no effective antidote and is freely available in the market [5].

AlP poisoning is a common mode of suicide in the agricultural community in northern India [6]. It has currently aroused interest with increasing number of cases in the past four decades due to increased use in agricultural and non-agricultural purpose, and also its easy availability has increased its misuse to commit suicide [7,8]. Unfortunately, it is rapidly becoming a very commonly used agent for self-poisoning in Iran [2,9-11].

### AlP forms

It is available as tablets (3 g, trade names: Phostoxin, Bhostoxin, Quickphos Phosphume Phostek) releasing 1 g PH_3_ or as pellets (0.6 g, Quickphos, Alphos, Cellphos). The tablets are green, brown or gray, and each tablet contains 56% AlP and 44% aluminum carbonate.

### The chemistry of AlP

The toxic effects of the AlP are due to deadly phosphine gas liberated when it reacts with water or hydrochloric acid in the stomach [4]. Phosphine gas (PH_3_) is the active pesticide component of AlP, which is rapidly absorbed by inhalation, ingestion, and skin or mucosal contacts [4]. AlP is available in the forms of tablets or pellets. It is the active component of the mixture so aluminum carbonate is added to prevent self-ignition of phosphine (PH_3_) which is liberated when AlP comes in contact with moisture. Each 3 g tablet releases 1 g of phosphine and so each 0.6 g pellet 0.2 g of phosphine gas on exposure to moisture and leaves behind a non-toxic grayish residue of aluminum hydroxide. In addition to these chemical properties, phosphine may spontaneously ignite in the presence of oxygen at concentrations above a threshold limit range of 0.48-1.9% (v/v) [12]. Phosphine gas is colorless and odorless but in exposure to air it may give a foul odor (garlicky or decaying fish) due to the presence of substituted phosphines and diphosphines [3]. Although exposures to toxicologically relevant concentrations of phosphine can occur in the absence of obvious odor detection [4].

### Mechanism of action

In the case of oral intake, the phosphine gas released is absorbed by the gastrointestinal tract with simple diffusion and is mainly excreted by the kidneys and lungs. Phosphine, like cyanide, inhibits mitochondrial cytochrome oxidase and cellular oxygen utilization [13-15].

Phosphine can inhibit cytochrome C oxidase in vitro but it has much less activity in vivo [16].

It can rapidly perturb mitochondrial conformation and inhibit oxidative respiration by 70%. This situation results a severe decrease in mitochondrial membrane potential [14].

In presence of AlP cellular superoxide and peroxide radicals are generated, with subsequent cellular damage by lipid peroxidation [17-20]. The higher levels of superoxide dismuatase (SOD), malonyldialdehyde (MDA) and catalase in post-mortems suggested their direct relation to mortality. Decreasing the serum levels of these biochemical parameters to a normal profiles in survivors by day 5 lead to a restriction of oxidative stress following phosphine elimination [21]. Some experimental results suggest that glutathione, melatonin, vitamin C and carotenes play an important role in reducing the effects caused by oxidative phosphine [8].

The direct toxic effects of phosphine on cardiac myocytes, fluid loss and adrenal gland can induce profound circulatory collapse [16]. Direct corrosive effects of phosphides and phosphine on body tissues have been reported [22].

### Toxicity

The fatal dose for a 70 kg adult is 150–500 mg [6,8]. Permissible exposure limit (PET) is 0.3 ppm over an 8 h shift (for factory stuffs). The range of short term exposure limit (STEL) is 1 ppm and immediate danger to life and health would be 200 ppm. For lethal dose in 30 min, the range of 400–600 ppm (10 mg/Kg AlP) has been determined. It has been reported that its LD50 in mice (inhalation of fumes) is 0.68 g/m^3^ during 65–75 min of exposure and for rats is 1.47 g/m^3^ during 35–50 min of exposure. LD50 for cats is 25 ppm (2–4 h daily during 3 days) [23].

### Etiology

AlP is the most common agent of poisoning in rural or sub-urban zones of some countries such as India, where it is usually ingested for suicide [5]. It is also uses as a suicide agent in Iran [24] but its poisoning in other countries may be due to occupational exposure [16]. AlP can induce rarely complications including hepatitis, acute tubular necrosis, gastroduodentitis, bleeding diathesis, corrosive like esophageal stricture and intravascular hemolysis [25-27].

### Epidemiology

An epidemiological study investigated health endpoints among Indian workers engaged in fumigation of stored grains [28]. The mean duration of employment among these workers was 11.1 years, and workplace concentrations of phosphine gas ranged from 0.17 to 2.11 parts per million (ppm) during the period of investigation. In this study, workers did not use facemasks, gloves or aprons as personal protective equipment. The most frequently reported symptoms reported immediately after completing fumigation activities included headache (31.8%), dyspnea (31.8%) and chest tightness (27.3%). Physical examinations of these workers were unremarkable, and no significant abnormalities were observed in tests of motor and sensory nerve conduction [28]. Outside of the workplace, phosphide fumigants are frequently implicated in association with epidemiological reports of accidental morbidity and mortality. In a one-year study period in Allahabad (India) out of a total 13,100 admissions, 301 suspected poisoning cases (2.3%) of the total admissions were recorded. The prevalence of AlP poisoning in this period was 11.7% and male to female ratio was 2:1. Age distribution study showed peak prevalence in the age group 11–30 years (65.1%) belonged to the rural area whereas 92 cases (30.6%) belonged to the urban areas [29].

A three-year (1999–2001) analysis of childhood poisonings in India found that 9% of all cases resulted from exposure to agricultural pesticides, and AlP fumigants were the most frequently implicated classification (29.67% of cases). Most of these cases involved ingestion pathways of exposure resulting from improper storage of fumigants [30]. Medical outcomes were not reported in this study. In another longitudinal, retrospective review of acute poisoning deaths in northern India, the AlP fumigants were identified as the most commonly implicated poison associated with fatalities (65% of cases) [31]. In this investigation, the incidence of cases resulting in death from the AlP increased during the period of observation (1972–1997). Morbidity and mortality have also been reported from phosphine inhalation in association with the in-transit fumigation of ships and railways [32,33]. In Iran, AlP poisoning has been reported frequently for suicide attept [34]. Unfortunately, the mortality rates were reported between 40 to 100%. In a four-year study period in northern Iran during 1997–2001, out of 1571 poisoning cases, 33 AlP poisoning cases (2.1%) were recorded [9] Another study in Gonbad (Iran), showed that out of 21 AlP poisoning cases, 19 cases have died. The mortality rate in this study was 90.5% [35]. Overall mortality rate of AlP poisoning was reported 31% in a retrospective 7 years study from 2000–2007 in Tehran [36]. Unpublished data from Babol town (Iran) showd 25 AlP poisoened cases out of total 1280 cases of poisoning during 2003–2009. In this study, 2 of 25 AlP posoning cases were intoxicated accidentaly and the others administered AlP for suicide. From nine cases of death, 7 were female. Age range was 15 to 70 with mean of 27.3. From 25 cases, 6 patients belonged to the rural area and 18 cases were from the urban sector. The ratio of male to female was 0.6 [37]. Although the percentage of poisoning with AlP is low, but the mortality rate of this kind of intoxication is very high. The mortality rate of AlP in some studies, were reported between 37-100% [34,38].

### Occupational exposure

Exposure to AlP is a common cause of poisoning from agricultural pesticide exposures in some parts of the world [30,31,39].

Chemicals like AlP, having a mortality rate more than 50% are easily available in the markets [40]**.** The epidemiology of occupational and environmental exposure to phosphine gas in association with phosphide fumigants has been reviewed by the National Institute of Occupational Safety and Health (NIOSH) [29]. Relevant findings in a 10-year study of 205 symptomatic exposures included the observation that 75% of cases occurred among individuals who were not directly handling or applying the fumigants. In 63% of cases among applicators, and 45% of cases among non-applicators, symptomatic exposure was reported to have occurred from the normal handling of phosphide fumigants (consistent with the product labeling). In this case series, approximately 25% of exposures to AlP among applicators occurred in the context of a misapplication (handling violations) [29]. There were no reported occupational cases occurring among individuals involved in the manufacturing process of phosphide fumigants in this series. A review of an epidemiological report demonstrates that occupational and environmental exposure to phosphine may also occur from scenarios that do not involve phosphide fumigants. Case reports of inhalation exposure to phosphine gas have been described in association with the clandestine synthesis of methamphetamine, as phosphine can be produced from phosphorous acid that is formed when iodine and red phosphorus are combined in aqueous media. Three fatalities have been reported from acute inhalation of phosphine gas, which occurred in a motel room where methamphetamine was being synthesized [41]. The other study described a case of transient symptoms of dizziness, headache, cough and diarrhea in a law enforcement official who was investigating a clandestine methamphetamine laboratory [42]. In this case, the affected individual was exposed to phosphine gas at a concentration of 2.7 ppb for 20–30 min [4].

### Clinical manifestations

Vomiting, abdominal pain, loose motions, restlessness have been observed. Cardiovascular complications include thread pulse, tachycardia, tachypnoea, acidosis, marked hypotension, palpitation, and unresponsive shock to conventional treatment. Patients remain mentally clear till cerebral anoxia due to shock supervenes resulting in drowsiness, delirium and coma [28]. Some common complications of AlP poisoning include hemorrhage, acute renal failure, disseminated intravascular coagulation and arrhythmias. Several ECG changes ranging from ST segment elevation/depression, PR and QRS interval prolongation, complete heart block to ectopic pace making and also fibrillation have been reported following ALP poisoning. Reversible myocardial injury has been described as well [28]. In the case of AlP poisoning patients can present anteroinferior wall ischaemia with incomplete right bundle branch block (RBBB) which is progressed to idioventricular rhythm, complete RBBB, and T-wave flattening simulating myocardial ischaemia. These changes were due to toxic injury to myocardium [43].

Pulmonary edema, dyspnoea, cyanosis, & altered sensorium may be discovered in AlP poisoning. Other rare effects include hepatitis, disseminated intravascular coagulation, and acute tubular necrosis. The complications noticed are pericarditis, congestive cardiac failure, acute gastrointestinal haemorrhage and acute respiratory arrest [6,44].

## Diagnosis

### Laboratory & clinical

Diagnosis is made by laboratory and clinical tests. The factors of positive history of ingestion, symptoms compatible with AlP ingestion and chemical test for phosphine positive in gastric aspirate and breath alone or in combination would help the diagnosis. The breath of AlP intoxicated patients has a garlic-like odor. The plan of diagnosis is based on the patient’s history and a positive result (blackening) on tests of the patient's breath with paper moistened with fresh silver nitrate solution due to exhalation of PH_3_ or by biochemical analysis of blood or gastric aspirate for phosphine [45-47].

Using filters impregnated with silver nitrate [48] and ion chromatographic methods [49] were recruited to determine phosphine in the bio-samples. Gas chromatographic procedure in survivors [50-52] and post-mortem specimens [53] has been developed for the measurement of phosphine levels.

Gas chromatographic technique using a nitrogen phosphorus detector has been introduced for phosphine measurement in post mortem stomach contents, blood, and liver specimens [54].

Laboratory assessment is mainly done to obtain the prognosis. Leucopenia indicates severe AlP toxicity. Increased serum glutamic oxaloacetic transaminase (SGOT) or serum glutamic pyruvic transaminase (SGPT) and induced metabolic acidosis indicate moderate to severe AlP overdose. Decreased plasma magnesium level has been reported while potassium might have been increased or decreased [55]. Plasma raised level of renin is significant as its level has a direct relationship with mortality and proportion to the dose of AlP. The serum level of cortisol is usually decreased in severe AlP poisoning [56].

It has been demonstrated that AlP can induce hepatotoxicity. The main findings were sinosoid conjestion, fatty liver changes, central vein conjestion, destruction of neucleos of hepatocytes and centrilubolar necrosis. Manifestations of hepatotoxicity usually develop 72 hours after AlP intoxication. Death due to acute hepatocellular toxicity and fulminant hepatic failure has also been reported in acute intoxication.

Cardiovascular and respiratory system changes can also lead to death [16,57]. In myocardial tissue, AlP induces conjestion, necrosis and leucocytes infiltration [38]. ECG shows various manifestations such as ST depression or elevation, bundle branch block, ventricular tachycardia, ventricular fibrillation [28,58-60]. Wall motion abnormalities, decreased ejection fraction, generalized hypokinesia of the left ventricle, and pericardial effusion can be seen in echocardiography [61].

Edema, congestion, hemorrhage, athlectasia, capilary vasodialation and alveolar wall thikening were the most pulmonary complications of AlP intoxication [38]. Chest X-ray may represent hilar or perihilar congestion, when ARDS occurs [58].

Patients may demonstrate hyperglycemia and metheomoglubinemia [24,62]. Development of refractory shock, ARDS, aspiration pneumonitis, anaemia, metabolic acidosis, electrolyte imbalance, coma, severe hypoxia, gastrointestinal bleeding, and pericarditis may be observed following acute AlP poisoning but these manifestations are associated with poor prognosis. The outcome of intoxication correlates best with the number of vomiting the patient gets after ingestion and the severity of hypotension the patient develops [44].

Simplified Acute Physiology Score II (SAPSII) was proposed to better estimating the outcome of paitients with acute AlP poisoning requiring ICU admission. Based on a study, SAPSII calculated within the first 24 hours was recognized as a good prognostic indicator [63].

### Postmortem findings

Biochemical and histopathological findings in post-mortem cases revealing pulmonary oedema, asphyxic lesions in the pulmonary parenchyma, gastrointestinal mucosal congestion, and petechial haemorrhages on the surface of liver and brain. Desquamation of the lining epithelium of the bronchioles, vacuolar degeneration of hepatocytes, dilatation and engorgement of hepatic central veins, sinusoids and areas showing nuclear fragmentation [64,65].

### Experimental study

Many studies were done to obtain possible antidote or treatment of AlP intoxication [44,66-68]. Magnesium content is decreased by AlP poisoning and it is believed that to compensate the magnesium levels can deteriorate the AlP poisoning outcomes [67,69]. AlP poisoning was intensity induced in mice to achieve an antidote [34]. In that study, male albino mice were intoxicated by AlP (10, 20 and 40 mg/kg) and its LD50, histopathological effects on heart, lung, kidney and liver were studied. The dose 40 mg/kg of AlP was considered as the LD50. The mice that were exposed to this dose died during 35 ± 15 min. Pathologic findings showed common microscopic changes in the liver. Trying to achieve an antidote, showed that also pretreatment of sodium selenite has not affected mortality latency, but it decreased the pulmonary and hepatic complications (Table 1). This result is consistent with previous studies [70,71]. N-acetyl systeine (NAC) in dose 50–100 mg/kg also improved the hepatic manifestations and prevented hepatic necrosis. This finding is in agreement to some other investigations [28,72]. The NAC also delayed the mortality latency time up to 138 ± 13 min. Vitamin C (500–1000 mg/kg) delayed mortality latency time up to 250 ± 70 min. Based on this study pretreatment of sodium selenite does not improve the mortality rate except the pathologic findings, but the NAC delays the mortality latency time and can prevent hepatic complications [34]. It is suggested that the NAC can be administered as an effective treatment in AlP poisoned patients. Since sodium selenite can inhibit free radicals production and prevent oxidative stress [73-75], should be considered as a possible treatment in the case of AlP poisoning.

**Table 1:** Mean ± SEM of the delayed time to death induced by AlP administration 30 minutes after saline or drugs treatment in mice

**Time (minutes) Treatment group**	**Delayed time to death before treatment**	**Delayed time to death after treatment**	**P value**	**95% CI**
**AlP (40 mg/kg of mice) + saline**	35 ± 15	39 ± 14	NS	-
**AlP + sodium selenite (3 mg/kg)**	42 ± 12	45 ± 16	NS	-
**AlP + NAC (50 mg/kg)**	45 ± 20	138 ± 13	0.0001	-37.3 , - 92.19
**AlP + Vitamin C (500mg/kg)**	46 ± 12	250 ± 17	0.0005	-38.1, -367.9

***AlP****: Aluminum phosphide;****NAC****: N-acetyl cysteine;****NS****: none significant by t-student test.*

Reproduced from *Moghadamnia, A.A., et al., Aluminum phosphide poisoning in mice and the procedure for its management. J Babol Univ Med Sci . 2000.****2****(4): p. 25-33.*

### Role of exposure route

The presenting symptoms depend on the route of administration. Poisoning by inhalation produces irritation of the mucous membrane, dizziness, easy fatigability, nausea, vomiting, headache and diarrhea in mild exposure. Ataxia, numbness, paraesthesia, muscle weakness, paralysis, diplopia and jaundice result from a moderate degree of exposure. In severe cases of inhalational poisoning, the patient presents with acute respiratory distress syndrome (ARDS), congestive cardiac failure, convulsion and coma. Nausea, vomiting and abdominal pain are the earliest symptoms that appear after ingestion. Gastrointestinal manifestations which present in moderate to severe intoxication are abdominal pain, epigastric tenderness and excessive thirst while cardiovascular abnormalities seen are profound hypotension, dry pericarditis, myocarditis, acute congestive heart failure and arrhythmias. Involvement of the respiratory system may lead to dyspnoea, which may progress to Type I or II respiratory failure. Nervous system manifestations include headache, dizziness, altered mental status, convulsion, acute hypoxic encephalopathy and coma. Renal and hepatic failures are the other manifestations. Some rare findings in AlP poisoning are muscular weakness, wasting, tenderness in proximal lower limb muscles, bleeding diathesis due to capillary damage, acute adrenocortical insufficiency and the pseudoshock syndrome due to impaired fluid distribution which results in micro-circulatory failure [76].

## Mortality

The specified fatal dose of AlP is 0.15-0.5 g. However, most of the patients present with ingestion of three or more tablets which invariably results in death [3]. The average time interval between ingestion of AlP and death is three hours (1–48 hours), 95% of the patients die within 24 hours and the commonest cause of death in this group is cardiac dysrhythmia. Severity of poisoning depends upon type of compound consumed. Fresh and active compounds (tablets) commonly affect heart, lungs, GI tract and kidneys; causing severe metabolic acidosis and high mortality. Broken or granular forms of tablets cause mild hypotension and ECG changes; mild metabolic acidosis and low mortality as activity of compound is less. Powder form of tablets is inactive; cause no systemic effects & no mortality [77]. Death after 24 hours is usually due to shock, acidosis, ARDS and cardiac dysrhythmia. Mortality depends upon dose of poison, severity of poisoning, duration of shock, failure of response of shock to resuscitative measures & severity of hypomagnesaemia [78]. Non-survivors have more severe hypotension and metabolic acidosis than the survivors who have more severe vomiting [44]. The mortality rate is highly variable, ranging from 37-100% and can reach more than 60% even in experienced and well equipped hospitals [3].

## Management

### General

Managing AlP poisoning, should be started as soon as possible. For confirmatory diagnosis getting history and clinical examination should not be delayed [79]. To reduce the absorption of phosphine gastric lavage with potassium permanganate (1:10,000) is done. Potassium permanganate (1:10000) is used as it oxidizes PH_3_ to form non-toxic phosphate. This is followed by slurry of activated charcoal (approximately 100 g) given through a nasogastric tube. Some cathartic (liquid paraffin) is given to accelerate the excretion of AlP and phosphine. Antacids and proton pump blockers are added for symptomatic relief [61,76]. Correction of plasma glucose level can help the patient to get better [24,79,80].

The most important factor for success is resuscitation of shock and institution of supportive measures as soon as the patient’s arrival. IV line should be established and 2–3 liters of normal saline should be given within the first 8–12 h guided by central venous pressure (CVP) and pulmonary capillary wedge pressure (PCWP). The aim is to keep the CVP at around 12–14 cm of water [81]. Some reports have recommended rapid infusion of saline (3–6 liters) in the initial 3 hr. 24 Low dose dopamine (4–6 μg/kg/min) is given to keep systolic blood pressure >90 mm Hg. Hydrocortisone 200–400 mg every 4–6 h is given intravenously to combat shock, reduce the dose of dopamine, check capillary leakage in lungs (ARDS) and to potentiate the responsiveness of the body to endogenous and exogenous atecholamines [10-12]. Oxygen is given for hypoxia. ARDS requires intensive care monitoring and mechanical ventilation. Phosphine excretion can be increased by maintaining adequate hydration and renal perfusion with intravenous fluids and low dose dopamine (4–6 μg/kg/min). Diuretics like furosemide can be given if systolic blood pressure is >90 mm Hg to enhance excretion as the main route of elimination of phosphine is renal [82]. All types of ventricular arrhythmias are seen in these patients and the management is the same as for arrhythmias in other situations [82]. Bicarbonate level less than 15 mEq/L requires sodabicarb in a dose of 50–100 mEq intravenously every 8 hour till the bicarbonate level rises to 18–20 mEq/L. Patients may require up to 300–500 ml of sodium bicarbonate [59]. Dialysis may be required for severe acidosis and acute renal failure [3].

### Specific therapy

The clinical management of intoxication from AlP is mainly supportive. The administration of H2 receptor antagonists has been recommended after AlP ingestion to reduce the gastric acidity and prevent further liberation of phosphine gas. Hypo-magnesemia is a common outcome of acute poisoning of phosphine gas exposure [66] and intracellular magnesium plays an important role as a co-factor in the synthesis and activity of glutathione and other antioxidants, the administration of intravenous magnesium has been investigated in cases of AlP poisoning. In a trial of 50 patients, individuals receiving repeated doses of intravenous magnesium had significant improvement in indicators of oxidative stress and a lower incidence of mortality (20%) in comparison to control subjects (44% mortality) [74]. Oral administration of the anti-ischemic drug trimetazidine, which works through a metabolic mechanism of decreasing the production of oxygen-derived free radicals and stimulating the oxidative metabolism of glucose [83,84] has been suggested. It was temporally associated with clinical improvement in a case report of occupational inhalation exposure to phosphine gas from AlP [85]. Magnesium sulphate use (both high and low dose) did not improve survival in controlled clinical trials. Hence using it is not recommended [86]. Administration of sorbitol solution (at a dose of 1-2 ml/kg) as a cathartic and vegetable oils and liquid paraffin as inhibitor of phosphine release from the overdosed AlP has been suggested [87]. It was reported that coconut oil has a role in managing acute AlP poisoning even 6 h post ingestion [80]. Digoxin has been suggested for treatment of cardiogenic shock induced by acute AlP intoxication [88].

## Conclusion

Exposure to phosphine gas released from ALP fumigants increases risks of major morbidity and mortality. There is an increasing need for improved knowledge of these risks with an emphasis on recognition, management and prevention. The fast progression to life-threatening symptoms, ineffective therapeutic ways to solve its intoxication and limited data on the efficacy of therapeutic interventions pose challenges to the clinicians and emergency staffs. People handling this fumigant must be aware of its lethal aspects. They should be prohibited from keeping and using this poison at the home. They should be advised to cover the tablets in open fields after use. They should keep their tablets away from the reach of children and other family members. Official health care system should restrict the open sales of this pesticide. Vendors and shop keepers should not sell the tablets to young people and children without proper verification and confirmation. Dealers not following the health system instructions should be punished. The manufacturers better be advised to make small packs of 2 – 3 tablets with suitable container. If possible all of the phosphide derivatives compounds should be forbidden forever for everyone.

## Competing interest

The author have no competing of interest.

## Authors’ contribution

AAM contributed solely to whole study and the paper.
